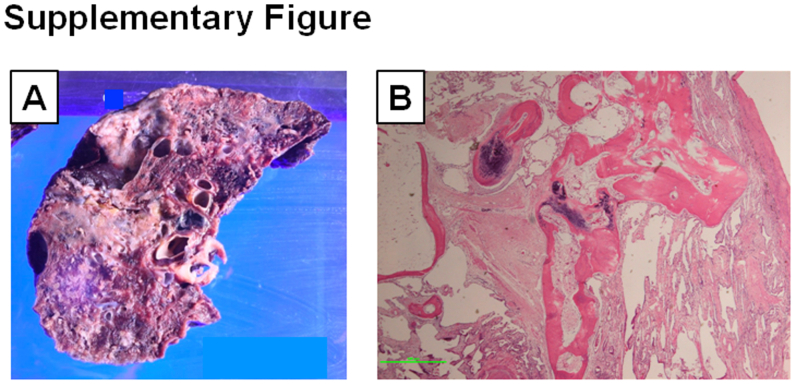# Lung Transplantation for Familial Diffuse Pulmonary Ossification

**DOI:** 10.1016/j.atssr.2024.02.015

**Published:** 2024-03-24

**Authors:** Akihiro Ohsumi, Yojiro Yutaka, Tomohiro Handa, Akihiko Yoshizawa, Hiroshi Date

**Affiliations:** 1 Department of Thoracic Surgery, Kyoto University Hospital, Kyoto, Japan; 2 Department of Advanced Medicine for Respiratory Failure, Kyoto University Hospital, Kyoto, Japan; 3 Department of Diagnostic Pathology, Kyoto University Hospital, Kyoto, Japan

## Abstract

We report 3 cases of extremely rare familial idiopathic diffuse pulmonary ossification, 2 of 3 received lung transplantation and the other is listed for lung transplantation. The clinical courses of family members varied greatly, and rapid deterioration could occur; therefore, early and close examination is recommended for transplant registration. During transplantation, the lungs appeared and felt exactly like a “pumice stone” and could not collapse, and good visual field was not easily obtained. Both patients had no recurrence of pulmonary ossification for more than 2 years.

Diffuse pulmonary ossification (DPO) is an uncommon condition characterized by the formation of mature bone fragments in the lung parenchyma. We report 2 cases of extremely rare familial DPO requiring lung transplantation (LTx). First, Patient 2, the father of this family was diagnosed as DPO, and listed for deceased-donor lung transplantation (DDLTx). The initial pretransplant history has been reported.^1^ Later, patient 1, the oldest son of patient 2, was also diagnosed as DPO and listed for DDLTx. The clinical course of patient 1 progressed rapidly, and received unilateral DDLTx prior to patient 2. Patient 2 received bilateral DDLTx subsequently.

## Case Reports

### Patient 1

**Figure 1:**
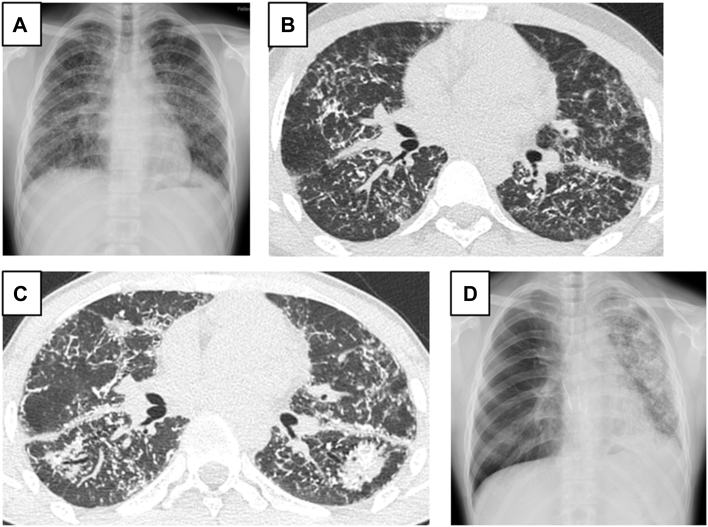
Chest radiography shows bilateral numerous dense nodules (A) and computed tomography shows multiple high-density nodules (B) at evaluation for listing. (C) Chest computed tomography before transplantation shows severe worsening of branching calcifications. (D) Chest radiography performed 35 months after transplantation shows progressive pulmonary ossification of the native lung.

**Figure 2:**
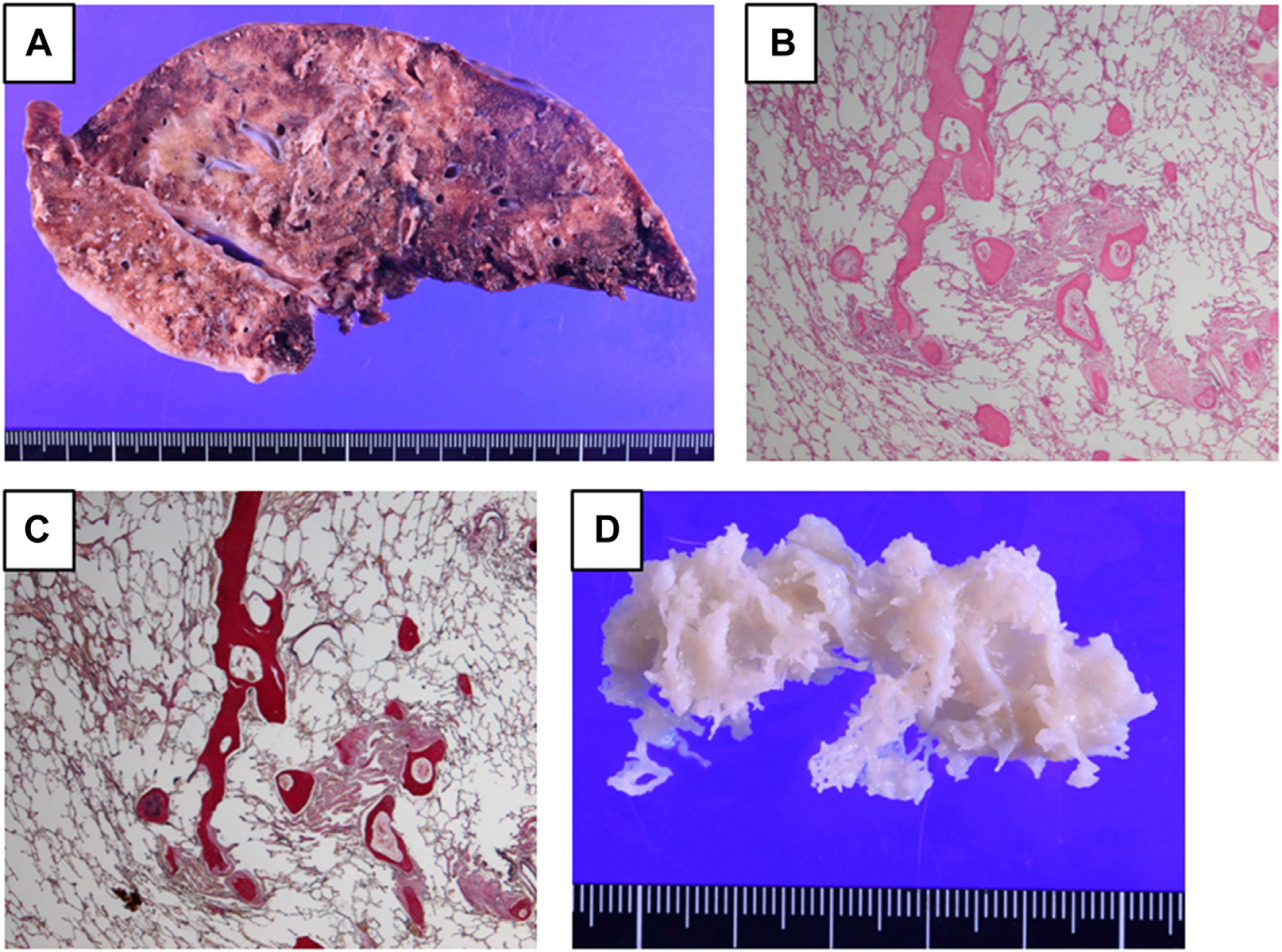
(A) Gross findings of the explanted lung fixed with formalin shows focal ossification with surrounding subpleural fibrosis. (B, C) Pathologic findings show intraalveolar ossification (B, hematoxylin and eosin staining; C, Elastica-Van Gieson staining, original magnification ×40). (D) Intraalveolar ossification lysed with hypochlorite showed “dendrite”.

The patient, the oldest son of patient 2, had cough and dyspnea on exertion at age 17 years. He was referred to the hospital. Although chest radiograph was almost clear 2 years before, it showed ground-glass opacities over all lung fields (Figure 1A), and chest computed tomography scan showed multiple high-density nodules (Figure 1B). The pulmonary function test demonstrated that percentage-predicted forced vital capacity (%FVC) decreased to 27.7% and percentage-predicted diffusion capacity of the lungs for carbon monoxide was reduced to 47.2%. The patient was diagnosed with DPO and was enlisted for DDLTx at age 18 years. The restrictive disorder deteriorated 16 months later; %FVC was 15.5%. Additionally, chest computed tomography showed severe worsening of calcifications (Figure 1C). The patient underwent right DDLTx 16 months after listing. The patient was anesthetized, and the peak pressure on the mechanical ventilator was >40 mm Hg. The right chest was opened with a hemiclamshell incision, and the appearance and tactile sensation of the lung were exactly “pumice stone.” The lung was difficult to collapse, and the visual fields were limited for surgical procedures. Then, sternum was split and venoarterial extracorporeal membrane oxygenation (ECMO) was established for sufficient visualization. The grafted lung was anastomosed and reperfused uneventfully, and the patient was weaned off ECMO. The patient’s postoperative course was uneventful, and he was discharged on postoperative day 48. The right transplanted lung progressed bronchiolitis obliterans without recurrence of DPO and the left native lung showed deteriorating ossification (Figure 1D); therefore the patient was evaluated for lung retransplantation 3 years after DDLTx. The explanted native lung fixed with formalin showed focal ossification with surrounding subpleural fibrosis (Figure 2A). Pathologic examination revealed multiple intraalveolar ossifications and fibrosis, consistent with DPO (Figures 2B, 2C). The intraalveolar fixed lung specimen showed dendritic ossification after hypochlorite lysis (Figure 2D).

### Patient 2

**Figure 3:**
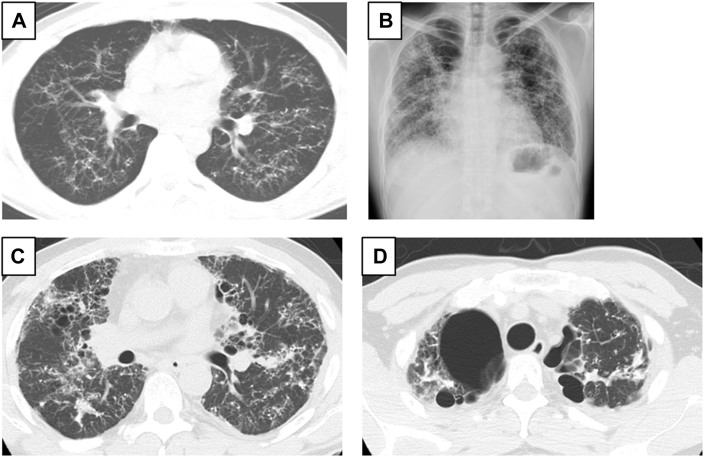
(A) Chest computed tomography images show numerous high-density nodules. (B) Chest radiograph showed worsening infiltration. Chest CT images shows increased micronodules (C) with bullous formation (D).

The patient was diagnosed with idiopathic dendriform DPO using thoracoscopic biopsy at age 30 yeras. Although chest imaging revealed bilateral high-density nodules (Figure 3A), pulmonary function tests revealed normal results at age 36 years. He presented at the hospital at age 46 years with cough and dyspnea on exertion, and %FVC dropped from 77.8% to 44.1%. He was considered for DDLTx and was enlisted. While waiting, the patient was treated with frequent pleurodesis for recurrent left pneumothorax. Chest radiography and computed tomography showed denser nodules with bulla formation (Figures 3B-3D). The patient underwent bilateral DDLTx at age 51 years. The chest was opened with a clamshell incision and bilateral pleural adhesions were observed. The lungs were like “pumice stone,” therefore the patient required high airway pressure ventilation. Additionally, pleural dissection was difficult because of pleurodesis. The left lung was slightly injured during pleural dissection, and the patient developed a bronchovenous fistula. Central venoarterial ECMO was established, then left lung ventilation was stopped and removed immediately. Left LTx was performed and reperfused, followed by right LTx. Finally, the patient showed no postoperative cardiac or neurologic complications.^2^ The posttransplantation course was uneventful, and the patient was discharged on postoperative day 64. The patient was in good health without recurrence of ossification 32 months after DDLTx. Gross findings of the lungs showed focal ossification, and pathologic examination revealed intraalveolar ossification and pleuroparenchymal fibroelastosis (Supplemental Figure A, B).

## Comment

Herein, we report the first case of familial DPO in patients who underwent DDLTx. DPO is an uncommon disease characterized by intrapulmonary mature bone formation, first reported by Luschka.^3^ DPO is uncommon in clinical practice and often a postmortem diagnosis due to subtle or nonspecific radiologic findings and clinical features.^3^ DPO has been classified into idiopathic and secondary forms.^4^ Idiopathic DPO is extremely uncommon.^1^^,^^5^ A nationwide retrospective study of idiopathic DPO in Japan showed that 2 of 22 cases were familial,^5^ and genetic abnormalities may be involved in pathogenesis. Therefore, when a patient is diagnosed with DPO, family members should be examined. Patient 2, the father, showed slow progress over 20 years, while for patient 1, his older son, pulmonary function deteriorated rapidly, necessitating listing within 1 year. The second son of patient 2, aged 19 years, was also diagnosed with DPO, and enlisted for DDLTx. Within the family, the father and his 2 sons were diagnosed as DPO requiring lung transplantation, and the clinical course seems to be quite different; therefore, early and close examination is recommended for listing.

DPO cases receiving DDLTx have been reported.^6^^,^^7^ Carney and associates^6^ reported that a patient’s pulmonary status progressively deteriorated over 2 years, and underwent bilateral LTx 3 years later. This is only the report which seemed to be primary DPO and was similar to patient 1. The native left lung of patient 1 deteriorated after LTx; therefore, bilateral LTx may be favorable for DPO. Additionally, patient 1 had bronchiolitis obliterans in the right transplanted lung, and was evaluated for lung retransplantation 3 years after DDLTx. Patient 2 is doing well 32 months after DDLTx. Both patients have had no signs of recurrence of DPO so far, suggesting that recipient’s humoral factors do not affect pulmonary ossification.

Some critical procedures for DPO must be considered. Regarding anesthesia, sufficient tidal volume could not be obtained even under high inspiratory pressure; therefore quick establishment of ECMO is mandatory. Regarding surgery, operative fields were limited because the patients with DPO had small chest cavities due to restrictive dysfunction, and osseous lungs like “pumice stone” cannot collapse. To obtain better operative fields, cessation of mechanical ventilation under ECMO and extension of hemiclamshell incision with splitting sternum will be helpful in unilateral LTx.

## Figures and Tables

**Supplementary Figure:**