# Supplementary material for: Lung Transplantation for Familial Diffuse Pulmonary Ossification
Source: Ann Thorac Surg Short Rep. 2024 Mar 24;2(3):495–8. doi: 10.1016/j.atssr.2024.02.015 (PMC11708718; doi:10.1016/j.atssr.2024.02.015)
Supplement: Supplemental figure legend [file mmc1.docx]

**Figure legends**

**Supplementary Figure**

Gross findings of the explanted lungs showed focal ossification (A). Pathologic findings showed intra-alveolar ossification and pleuroparenchymal fibroelastosis (B, hematoxylin and eosin staining; magnification ×200).
